# The Social Patterns of Age Discrimination: An Analysis of the Macro-Perspective-Interpretations

**DOI:** 10.1093/geroni/igaa057.1049

**Published:** 2020-12-16

**Authors:** Stefan Hopf

**Affiliations:** National University of Ireland Galway, Galway, Ireland

## Abstract

Modern societies can be regarded as service economies, consequently accessing services is an essential part of social and economic participation. Direct and indirect indiscrimination act as barriers to accessing and using services and one way to address these barriers is to implement anti-discrimination legislation and policy. From a sociological point of view, such policies and legal frameworks can be described as elements of the social discourse in these areas. These texts, along with their implicit and explicit interpretations of the problem, represent the official and legitimised stake of the socially available stock of knowledge of what constitutes age discrimination. Hence the shape and contribute to the general understanding of age discrimination. The study aims to investigate the interpretation patterns offered by the “supply” side, that is by those actors who in their work refer to but also (re-) shape and disseminate the problem interpretation contained in the official texts. To address this aim, focus groups with stakeholders and semi-structured interviews with legal and policy experts were conducted in Austria and Ireland. The findings highlight that experts and stakeholders’ definitions of age discrimination usually extend past legal and policy concepts. The expert and stakeholder approaches differ in their starting points for describing the problem, ranging from vulnerability considerations to human rights-based concepts and more structurally orientated needs-based criteria. Finally, the analysis also reveals a central distinguishing feature of age discrimination, namely the “de-temporalization” and “de-historicization” of the person, which is of equal importance as the de-individualization as a consequence of stereotyping

